# Pre-pregnancy BMI and weight gain: where is the tipping point for preterm birth?

**DOI:** 10.1186/1471-2393-13-120

**Published:** 2013-05-24

**Authors:** Saba W Masho, Diane L Bishop, Meaghan Munn

**Affiliations:** 1 Department of Epidemiology and Community Health and Obstetrics and Gynecology, Virginia Commonwealth University, P.O. Box 980212, Richmond, VA 23298-0212, USA; 2 The VCU Institute of Women’s Health, Virginia Commonwealth University, Richmond, VA, USA; 3 The VCU Center on Health Disparities, Virginia Commonwealth University, Richmond, VA, USA

## Abstract

**Background:**

Obesity in pregnant women is a major problem affecting both the mother and her offspring. Literature on the effect of obesity on preterm birth is inconsistent and few studies have investigated the influence of weight gain during pregnancy. This study examined the effect of maternal pre-pregnancy BMI and weight gain during pregnancy on preterm birth.

**Methods:**

Data from the Collaborative Perinatal Project (CPP) on 45,824 pregnant women with singleton, live-born infants with no sever congenital anomalies was analyzed. Primary outcome variables included preterm (< 37 weeks of gestation), categorized into spontaneous preterm with and without premature rupture of membrane (PROM) and indicated preterm. Maternal BMI was categorized into underweight (BMI < 18.50), normal weight (BMI =1 8.50 – 24.99), overweight (BMI = 25.00 – 29.99), and obese (BMI ≥ 30.00). Multinomial regression analysis was conducted and OR and 95% CI were calculated.

**Results:**

The rate of spontaneous preterm birth with PROM among overweight women decreased with increasing weight gain but increased among women who had excessive weight gain. Similarly, a U-shaped rate of spontaneous preterm birth with and without PROM was observed in obese women. Gaining less weight was protective of spontaneous preterm with and without PROM among overweight and obese women compared to normal weight women. Among underweight women, gaining < 7 kg or 9.5-12.7 kg was associated with increased odds of indicated preterm birth. Appreciable differences were also observed in the association between pre-pregnancy BMI, gestational weight gain and the subtypes of preterm births among African Americans and Caucasian Americans.

**Conclusion:**

Reduced weight gain during pregnancy among overweight and obese women is associated with reduced spontaneous preterm birth with and without PROM. Health care professionals and public health workers should be aware of this risk and adhere to the 2009 IOM guideline that recommended reduced weight gain during pregnancy for obese and overweight women.

## Background

Obesity is a major public health problem in the United States [1]. According to the 2007–08 NHANES survey, one in every three women in the United States is obese [2]*.* The rate of obesity was highest among non-Hispanic blacks at 50% [2,3]. Maternal obesity or obesity during pregnancy is of particular concern due to its adverse consequences on both the mother and her offspring [4-10]. However, the evidence demonstrating an association between maternal obesity and preterm birth (PTB) is less conclusive [4-6,11-18]. A recent meta-analysis provided evidence that the association between obesity and preterm birth may vary depending on the sub-type of preterm birth. The study reported being overweight (Body Mass Index (BMI) = 25–29.9) and obese (BMI = 30–34.9) to be protective of spontaneous preterm birth. However, it demonstrated an increased risk of preterm birth among obese II (BMI = 35–40 and Obese III (BMI = > 40) women. Further, the study reported no associated between premature rupture of membranes and high BMI [19].

In general, preterm birth is classified into spontaneous with premature rupture of membrane (PROM), spontaneous without PROM and indicated preterm birth. The latter is usually performed due to maternal or fetal problems. The etiology and risk factors of preterm births differ by the type of preterm birth [20,21]. Pre-pregnancy weight and gestational gain during pregnancy are also potential risk factors that may differentially affect these distinct types of preterm births. A study by Rudra et al. reported that pre-pregnancy over weight is associated with indicated preterm birth. However, the study reported a weaker association between spontaneous preterm birth and PROM [22]. The effect of obesity by types of preterm birth was also examined in a study that analyzed a large cohort of US women that reported associations between obesity and decreased risk of spontaneous preterm birth without PPROM and increased risk of PPROM [23].

Another factor that plays a significant role in the association between pre-pregnancy weight and preterm birth is race. A study from Florida that analyzed 540,981 birth cohorts reported that African American women were disproportionately affected by obesity and preterm births. While the odds of preterm births were 71% higher in African American women, the odds were only 15% higher in Caucasian obese women compared to normal weight women [24]. Unlike the above study, a recent case control study reported increased odds of preterm birth in obese Caucasian women but the inverse association (protective effect) was reported in obese African American women [25]. The inconsistencies in these findings are not clear; however the difference in study methodology may be an explanation for the differences reported.

The Institute of Medicine (IOM) and the public health community has been concerned with the increased trends of obesity [26]. In 2009, the IOM released an updated guideline on recommended weight gain during pregnancy in accordance to maternal pre-pregnancy BMI. The IOM report also identified major research gaps, including the impact of weight gain during pregnancy on maternal and child health outcomes [26]. Despite the plethora of literature [11,17,22,27-34], only few studies have examined the impact of pre-pregnancy obesity and weight gain during pregnancy on preterm births [22,27,28]. The majority of these studies were conducted when the obesity epidemic was increasing or at its peak when co-morbidities, lifestyle behaviors and stressors may have confounded the association. This analysis seeks to understand the influence of pre-pregnancy BMI and weight gain during pregnancy on preterm birth using a large prospective multisite data collected prior to the beginning of the obesity epidemic. Additionally, the data allowed examination of the different types of preterm births and the interaction by race, and of multiple factors that potentially impact this association, reflecting on the 2009 IOM recommended pregnancy weight gain guideline.

## Methods

Data for this analysis were drawn from the National Institute of Neurological and Communicative Disorders and Stroke (NINCDS) Collaborative Perinatal Project (CPP). The study methodology including, setting, population and recruitment has been described in detail elsewhere [35]. Briefly, the CPP was a multisite prospective cohort study that enrolled pregnant women at their first prenatal visit between 1959 and 1965 from 12 university-affiliated medical centers in the United States. These medical centers served predominantly African American, low income, and inner city populations. The recruitment sites were: Boston, Massachusetts; Buffalo, New York; New Orleans, Louisiana; New York, New York; Baltimore, Maryland; Richmond, Virginia; Minneapolis, Minnesota; Portland, Oregon; Philadelphia, Pennsylvania; Providence, Rhode Island; Memphis, Tennessee. The CPP gathered detailed information on the mother’s medical history, pregnancy, labor and delivery, as well as the children [36]. This secondary analysis is reviewed by the VCU Institutional Review Board for ethical standard.

Data was downloaded via File Transfer Protocol (FTP) from the Johns Hopkins University Bloomberg School of Public Health [36]. The dataset included information on all pregnancies and mother-child pairs (N = 59,391). Pregnant women with multiple births or missing plurality (N = 3,651), severe congenital anomalies at birth (N = 1,073), or non-live births, including abortion, molar pregnancies, stillbirths, and unknown etiology fetal deaths (N = 2,093), were excluded from this analysis. Additionally, a total of 6,750 mother-child pairs with data missing from one or more of the following study variables were also excluded: pre-pregnancy BMI (N = 4,497), gestational weight gain (N = 3,573), labor (N = 468), or gestational age (N = 212). This resulted in the inclusion of 45,824 pregnant women who delivered singleton, live-born infants with no major congenital anomalies.

The dataset included information on reproductive history including gravidity, parity, previous abortions, diabetes, preeclampsia, chronic hypertension, previous pregnancy outcomes, number of prenatal visits and prior history of low birth weight and preterm births. Current pregnancy history including delivery method, child’s sex, APGAR score, birth weight, gestational age, congenital anomalies, preeclampsia, gestational diabetes and hypertension, anemia and other pregnancy outcomes were collected. It also included data on previous medical history including history of diabetes, chronic hypertension, heart, kidney, liver and other chronic and acute diseases. Additionally, the dataset included lifestyle factors such as cigarette smoking, substance use during pregnancy and demographic information such as mother’s age, marital status, education, race, and socio-economic index.

The main outcome variable, gestational age, was provided in the dataset; and was calculated by subtracting the last menstrual period from the date of delivery. Gestational age was dichotomized into preterm birth (<37 weeks of gestation) and full term birth (≥37 weeks of gestation. In accordance to the literature, preterm was further categorized as: a) spontaneous with premature rupture of membranes, b) spontaneous without PROM, and c) indicated. In accordance to the literature, preterm was further categorized as: a) spontaneous with premature rupture of membranes, b) spontaneous without PROM, and c) indicated (induced). These subtypes of preterm birth were created using two variables, ‘labor onset type’ and ‘rupture of membrane reason’. ‘Labor onset type’ was categorized in the data as “spontaneous”, “induced”, or “none”. The ‘rupture of membrane reason’ was dichotomized as “spontaneous”, and “non-spontaneous”. The spontaneous preterm birth with PROM was coded ‘yes’, if the response for ‘Labor onset’ is ‘spontaneous’, ‘the rupture of membrane’ variable was coded ‘spontaneous’ and the gestational age at birth was <37 weeks. Conversely, the spontaneous preterm birth without PROM was coded ‘yes’, if the response for ‘Labor onset’ was ‘spontaneous’ and the rupture of membrane variable was coded ‘non-spontaneous’ and the gestational age at birth was <37 weeks. Finally, if the response to ‘Labor onset’ was ‘induced’ and the gestational age at birth was <37 weeks, this group was categorized as indicated preterm births.

Maternal BMI was calculated using self-reported pre-pregnancy weight and height. Pre-pregnancy BMI was categorized according to the World Health Organization’s definition into four groups: underweight (BMI < 18.50), normal weight (18.50 – 24.99), overweight (25.00 – 29.99), and obese (BMI ≥ 30.00) [37]. Pregnancy weight gain was determined using the mother’s weight just prior to delivery subtracted from the pre-pregnancy weight. Pregnancy weight gain was categorized into quartiles (< 7.0 kg, 7.0-9.4 kg, 9.5-12.7 kg, and >12.7 kg).

Maternal hypertension was defined as hypertension documented by evidence occurring during any part of the pregnancy (including before 24 weeks) or a diagnosis of chronic hypertension. Preeclampsia was categorized as “yes” if mild or severe preeclampsia or eclampsia was indicated in the dataset (preeclampsia diagnosis was obtained from medical history). Prior and current medical conditions and pregnancy outcomes were categorized as ‘yes’ and ‘no’. Lastly, the number of prenatal visits was examined and categorized as ‘1-5’, ‘6-10’,’11-14’ and’15 or more’. Due to insufficient data, the Kotelchuck index for adequacy of pregnancy was not calculated.

Cigarette smoking and other substance use during pregnancy, were coded as ‘yes’ or ‘no’. Maternal race was collapsed into three categories: White, Black, and Other. The ‘other’ category included “Asian”, “Puerto Rican”, and “Other”. Maternal education was categorized as less than high school, high school, or greater than high school attainment. Socio-economic index, a computed variable that was provided in the data set was examined. The variable was calculated by averaging the component scores for education, occupation, and family income. A lower score indicates lower affluence and conversely, higher indices indicate greater affluence (possible range of 0.0 to 10.0) [38].

Data were examined using descriptive statistics, including frequencies, means and standard deviations, chi-square and t-tests. Multinomial logistic regression was conducted to investigate the association between pre-pregnancy BMI and the different sub-types of preterm categories stratifying by weight gain during pregnancy and to control for the effect of confounders. Odds ratios and 95% confidence intervals were calculated and the most efficient model was determined using 10% change in the estimate. Statistical analyses were performed with the SAS software (version 9.3; SAS Institute Inc., Cary, NC).

## Results

The average age of the study sample was 24.1 years (standard deviation (SD) = 6.0 years) (Table 1). The racial distribution consisted of a slightly higher proportion of Blacks (48.5%) than Whites (44.4%). The majority reported to be married (76.2%) and having less than high school education (56.9%). The majority of births were to multiparous women (71.2%) who delivered full term (86.1%) infants. However, nearly 8% had spontaneous preterm birth with PROM, 5% delivered a spontaneous preterm birth without PROM, and 1.2% of preterm births were indicated. Over two-thirds of the study population had normal pre-pregnancy weight (68.9%); and 15% and 6% were overweight and obese, respectively.

**Table 1:** Characteristics of the study population by BMI categories

**Characteristics**	**Total (N = 45,824)**	**Underweight BMI <18.50 N = 4,348 (9.5%)**	**Normal BMI 18.50-24.99 N = 31,555 (68.9%)**	**Overweight BMI 25.00-29.99 N = 6,961 (15.2%)**	**Obese BMI ≥ 30.00 N = 2,960 (6.5%)**	
	**N (%)**	**N (%)**	**N (%)**	**N (%)**	**N (%)**	**p-value**
Mean Age (SD)	24.1 (6.0)	22.4 (5.2)	23.4 (5.7)	26.3 (6.5)	28.1 (6.6)	<0.0001
Marital						<0.0001
Single	7075 (15.4)	794 (18.3)	5089 (16.1)	874 (12.6)	318 (10.7)	
Married	34913 (76.2)	3220 (74.1)	24004 (76.1)	5416 (77.8)	2273 (76.8)	
Other	3834 (8.4)	334 (7.7)	2460 (7.8)	671 (9.6)	369 (12.5)	
Missing	2 (0.0)	0 (0.0)	2 (0.0)	0 (0.0)	0 (0.0)	
Education						<0.0001
Less than High School	26066 (56.9)	2528 (58.1)	17193 (54.5)	4329 (62.2)	2016 (68.1)	
High School	13753(30.0)	1311 (30.2)	9662 (30.6)	2019 (29.0)	761 (25.7)	
More than High School	5204 (11.4)	432 (9.9)	4146 (13.1)	491 (7.1)	135 (4.6)	
Missing	801(1.8)	77 (1.8)	554 (1.8)	122 (1.8)	48 (1.6)	
Race						<0.0001
White	20341 (44.4)	2009 (46.2)	14768 (46.8)	2568 (36.9)	996 (33.7)	
Black	22231 (48.5)	1989 (45.8)	14538 (46.1)	3896 (56.0)	1808 (61.1)	
Other	3071 (6.7)	296 (6.8)	2126 (6.7)	495 (7.1)	154 (5.2)	
Missing	181 (0.4)	54 (1.2)	123 (0.4)	2 (0.0)	2 (0.1)	
SES Index						<0.0001
0.0-3.9	17850 (39.0)	1803 (41.5)	11619 (36.8)	3016 (43.3)	1412 (47.7)	
4.0-5.9	13967 (30.5)	1295 (29.8)	9371 (29.7)	2033 (32.8)	998 (33.7)	
6.0-9.5	12830 (28.0)	1138 (26.2)	9764 (30.9)	1456 (20.9)	472 (16.0)	
Missing	1177 (2.6)	112 (2.6)	801 (2.5)	186 (2.7)	78 (2.6)	
Tobacco use	21154 (46.2)	2257 (51.9)	14819(47.0)	2907 (41.8)	1171 (39.6)	<0.0001
Missing	280 (0.6)	23 (0.5)	192 (0.6)	39 (0.6)	26 (0.9)	
Substance use	50 (0.1)	1 (0.0)	43 (0.1)	5 (0.1)	1 (0.0)	0.2416
Missing	283 (0.6)	24 (0.6)	195 (0.6)	45 (0.7)	19 (0.6)	
Parity						<0.0001
Primiparous	13028 (28.4)	1567 (36.0)	10010 (31.7)	1121 (16.1)	330 (11.2)	
Multiparous	32635 (71.2)	2758 (63.4)	21439 (67.9)	5816 (83.6)	2622 (88.6)	
Missing	161 (0.4)	23 (0.5)	106 (0.3)	24 (0.3)	8 (0.3)	
Previous Premature infant						<0.0001
Previous Preterm birth	7602 (16.6)	874 (20.1)	4915(15.6)	1218 (17.5)	595 (20.1)	
Previous Full-term birth	25054 (54.7)	1885 (43.4)	16537 (52.4)	4604 (66.1)	2028 (68.5)	
No Previous births	13067 (28.5)	1577 (36.3)	10037 (31.8)	1122 (16.1)	331 (11.2)	
Missing	101 (0.2)	12 (0.3)	66 (0.2)	17 (0.2)	6 (0.2)	
Number of Prenatal Visits						<0.0001
1-5	10158 (22.2)	1016 (23.4)	6903 (21.9)	1514 (21.8)	725 (24.5)	
6-9	16326 (35.6)	1611 (37.1)	11171 (35.4)	2495 (35.8)	1049 (35.4)	
10-14	15854 (34.6)	1445 (33.2)	11036 (35.0)	2428 (34.8)	945 (31.9)	
15 and above	3446 (7.5)	270 (6.2)	2421 (7.7)	515 (7.4)	240 (8.1)	
Missing	40 (0.1)	6 (0.1)	24 (0.1)	9 (0.1)	1 (0.0)	
Diabetes	223 (0.5)	15 (0.3)	97 (0.3)	54 (0.8)	57 (1.9)	<0.0001
Missing	256 (0.6)	28 (0.6)	173 (0.6)	37 (0.5)	18 (0.6)	
Hypertension	2172 (4.7)	101 (2.3)	1062 (3.4)	503 (7.2)	506 (17.1)	<0.0001
Missing	94 (0.2)	5 (0.1)	67 (0.2)	14 (0.2)	8 (0.3)	
Anemia	9153 (20.0)	980 (22.5)	6366 (20.2)	1323 (19.0)	484 (16.4)	<0.0001
Missing	222 (0.5)	21 (0.5)	152 (0.5)	34 (0.5)	15 (0.5)	
Preelampsia	6380 (14.0)	425 (9.8)	3971 (12.6)	1216 (17.5)	768 (26.0)	<0.0001
Missing	65 (0.1)	4 (0.1)	44 (0.1)	11 (0.2)	6 (0.2)	
Weight gain (kg)						<0.0001
< 7.0	12863 (28.1)	767 (17.6)	7851 (24.9)	2651 (38.1)	1594 (53.9)	
7.0-9.4	9254 (20.2)	905 (20.8)	6721 (21.3)	1234 (17.7)	384 (13.3)	
9.5-12.7	12270 (26.8)	1387 (31.9)	9046 (28.7)	1447 (20.8)	390 (13.2)	
> 12.7	11437 (25.0)	1289 (29.7)	7937 (25.2)	1629 (23.4)	582 (19.7)	
Delivery method						<0.0001
Vaginal	43422 (94.8)	4171 (95.9	30073 (95.3)	6506 (93.5)	2672 (90.3)	
Cesarean	2401 (5.2)	177 (4.1)	1481 (4.7)	455 (6.5)	288 (9.3)	
Missing	1 (0.0)	0 (0.0)	1 (0.0)	0 (0.0)	0 (0.0)	
Birth weight						<0.0001
Low (<2500 grams)	4645 (10.1)	713 (16.4)	3259 (10.3)	478 (6.9)	195 (6.6)	
Normal (≥ 2500 grams)	41151 (89.8)	3633 (83.6)	28282 (89.6)	6477 (93.1)	2759 (93.2)	
Missing	28 (0.1)	2 (0.1)	14 (0.0)	6 (0.1)	6 (0.2)	
Gestational Age						<0.0001
Spontaneous PTB with PROM	3526 (7.7)	384 (8.8)	2421 (7.7)	508 (7.3)	213 (7.2)	
Spontaneous PTB no PROM	2278 (5.0)	279 (6.4)	1595 (5.1)	283 (4.1)	121 (4.1)	
Indicated PTB	557 (1.2)	48 (1.1)	381 (1.2)	80 (1.2)	48 (1.6)	
Full Term Birth	39463 (86.1)	3637 (83.7)	27158 (86.1)	6090 (87.5)	2578 (87.1)	

**Figure 1:**
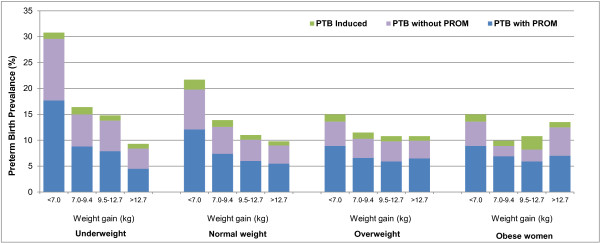
Prevalence of preterm birth by pre-pregnancy BMI and pregnancy weight gain.

Figure 1 displays the prevalence rate of the preterm birth sub-types by pre-pregnancy BMI and weight gained during pregnancy. Among underweight and normal weight pregnant women, the rates of all sub-types of preterm births decreased with increased gestational weight. On the other hand, in overweight women, the rate of preterm birth with PROM showed a reversed J-shape with increasing weight gain. Similarly, among obese women, the rates of all sub-types of preterm births were highest among those who gained the lowest (<7.0 kg) and highest weight categories for spontaneous preterm birth with and without PROM.

Table 2 shows the unadjusted association between obesity, gestational weight gain and preterm birth. Gaining less weight (< 7.0 kg) was significantly associated with increased odds of spontaneous preterm birth with and without PROM in underweight women. However, gaining less weight (< 7.0 kg) was associated with decreased odds of all types of preterm birth in overweight women and spontaneous preterm birth with and without PROM in obese women. Gaining 7.0-9.4 kg was statistically associated with spontaneous preterm birth without PROM in obese women compared to normal weight women. Additionally, gaining 9.5-12.7 kg was statistically associated with increased odds of spontaneous preterm birth with and without PROM and indicated preterm birth in underweight and obese women, respectively. The odds of spontaneous preterm birth without PROM were higher among obese women who gained >12.7 kg compared to normal weight women.

**Table 2:** Association between pre-pregnancy weight, weight gain during pregnancy and preterm birth – unadjusted analysis

	**Underweight**			**Overweight**			**Obese**		
	**Spontaneous births**		**Induced**	**Spontaneous births**		**Induced**	**Spontaneous births**		**Induced**
**Weight gain (kg)**	**PTB with PROM**	**PTB without PROM**	**PTB**	**PTB with PROM**	**PTB without PROM**	**PTB**	**PTB with PROM**	**PTB without PROM**	**PTB**
**< 7.0**	1.66***	1.75***	0.72	0.70***	0.57***	0.68*	0.58***	0.54***	0.87
	(1.36, 2.03)	(1.38, 2.21)	(0.37, 1.42)	(0.58, 0.79)	(0.46, 0.69)	(0.47, 0.98)	(0.47, 0.70)	(0.42, 0.69)	(0.58, 1.30)
**7.0-9.4**	1.22	1.23	1.13	0.86	0.68*	0.90	0.88	0.38*	0.74
	(0.96, 1.57)	(0.92, 1.65)	(0.63, 2.03)	(0.67, 1.09)	(0.50, 0.94)	(0.52, 1.57)	(0.59, 1.32)	(0.19, 0.76)	(0.27, 2.03)
**9.5-12.7**	1.39*	1.52**	1.17	1.00	0.97	1.14	0.99	0.57	2.82*
	(1.12, 1.73)	(1.19, 1.95)	(0.60, 2.06)	(0.79, 1.26)	(0.73, 1.29)	(0.66, 1.99)	(0.64, 1.52)	(0.29, 1.11)	(1.45, 5.49)
**>12.7**	0.82	1.11	1.11	1.19	1.00	1.05	1.35	1.65*	1.29
	(0.62, 1.09)	(0.82, 1.51)	(0.60, 2.07)	(0.96, 1.49)	(0.75, 1.34)	(0.59, 1.87)	(0.97, 1.88)	(1.13, 2.41)	(0.56, 3.00)

*p < 0.05.

**p < 0.001.

***p < 0.0001.

Stratified analysis by race showed underweight women who gained less weight (<7.0 kg) had increased odds of spontaneous preterm birth with and without PROM, regardless of their race (Table 3). Conversely, gaining >12.7 kg was associated with decreased odds of spontaneous preterm birth with PROM in underweight women compared to normal weight women. Overweight and obese women who gained less weight (<7.0 kg) had decreased odds of spontaneous preterm birth with and without PROM regardless of their race. While no difference was observed in Whites, overweight and obese Black women who gained 7.0-9.4 kg had lower odds of spontaneous preterm birth without PROM compared to normal weight women. Similarly, gaining 9.5-12.7 kg in Black obese women was associated with lower odds of spontaneous preterm birth without PROM compared to normal weight women.

**Table 3:** Association between pre-pregnancy weight, weight gain during pregnancy and preterm birth – stratified by race

	**Underweight**			**Overweight**			**Obese**		
**Weight gain (kg)**	**Spontaneous births**		**Induced**	**Spontaneous births**		**Induced**	**Spontaneous births**		**Induced**
	**PTB with PROM**	**PTB without PROM**	**PTB**	**PTB with PROM**	**PTB without PROM**	**PTB**	**PTB with PROM**	**PTB without PROM**	**PTB**
**White**									
**< 7.0**	1.96**	2.13*	0.57	0.64*	0.34**	0.65	0.44**	0.36*	0.60
	(1.34, 2.86)	(1.33, 3.41)	(0.21, 1.58)	(0.46, 0.90)	(0.19, 0.60)	(0.38, 1.10)	(0.27, 0.71)	(0.18, 0.72)	(0.31, 1.16)
**7.0-9.4**	1.28	1.93*	1.18	0.90	1.39	0.73	0.57	0.38	1.47
	(0.80, 2.02)	(1.13, 3.31)	(0.56, 2.50)	(0.55, 1.47)	(0.79, 2.45)	(0.31, 1.70)	(0.18, 1.83)	(0.05, 2.72)	(0.45, 4.76)
**9.5-12.7**	2.04**	2.41***	1.45	1.00	0.38	0.89	1.04	0.79	2.22
	(1.39, 2.98)	(1.56, 3.73)	(0.70, 2.98)	(0.59, 1.69)	(0.14, 1.04)	(0.35, 2.24)	(0.38, 2.86)	(0.19, 3.26)	(0.68, 7.23)
**> 12.7**	1.36	1.51	1.60	1.23	0.81	0.76	1.83	1.62	-
	(0.27, 2.16)	(0.87, 2.64)	(0.76, 3.35)	(0.71, 2.12)	(0.39, 1.71)	(0.27, 2.16)	(0.79, 4.27)	(0.58, 4.51)	
**Black**									
**< 7.0**	1.51*	1.70**	0.84	0.62***	0.54***	0.76	0.59***	0.50***	1.17
	(1.17, 1.95)	(1.27, 2.28)	(0.30, 2.34)	(0.51, 0.74)	(0.43, 0.68)	(0.44, 1.32)	(0.44, 0.70)	(0.37, 0.67)	(0.66, 2.06)
**7.0-9.4**	1.03	1.02	1.24	0.76	0.41***	1.30	0.68	0.30*	0.36
	(0.74, 1.42)	(0.71, 1.49)	(0.48, 3.21)	(0.56, 1.01)	(0.27, 0.63)	(0.61, 2.77)	(0.43, 1.07)	(0.14, 0.65)	(0.05, 2.68)
**9.5-12.7**	1.18	1.26	1.09	0.82	0.96	1.31	0.82	0.41*	3.25
	(0.90, 1.56)	(0.90, 1.75)	(0.42, 2.81)	(0.62, 1.09)	(0.70, 1.33)	(0.60, 2.86)	(0.51, 1.33)	(0.18, 0.93)	(1.33, 7.90)
**> 12.7**	0.66*	1.10	0.65	0.97	0.90	1.14	0.97	1.31	1.50
	(0.46, 0.97)	(0.74, 1.62)	(0.20, 2.15)	(0.76, 1.26)	(0.65, 1.26)	(0.54, 2.40)	(0.67, 1.40)	(0.86, 1.99)	(0.58, 3.87)

***p < 0.0001.

**p < 0.001.

*p < 0.05.

The adjusted model, controlling for study sites, maternal education, and race is shown in Table 4.

**Table 4:** Association between pre-pregnancy weight, weight gain during pregnancy and preterm birth – adjusted analysis

**Weight gain (kg)**	**Underweight**			**Overweight**			**Obese**		
	**Spontaneous births**		**Induced**	**Spontaneous births**		**Induced**	**Spontaneous births**		**Induced**
	**PTB with PROM**	**PTB without PROM**	**PTB**	**PTB with PROM**	**PTB without PROM**	**PTB**	**PTB with PROM**	**PTB without PROM**	**PTB**
**< 7.0**	1.70***	1.74***	0.79	0.61***	0.51***	0.68*	0.53***	0.50***	0.84
	(1.38, 2.09)	(1.36, 2.22)	(0.40, 1.57)	(0.52, 0.71)	(0.42, 0.62)	(0.47, 0.99)	(043, 0.65)	(0.39, 0.64)	(0.56, 1.27)
**7.0-9.4**	1.19	1.18	1.23	0.72*	0.58**	0.91	0.66*	0.28**	0.77
	(0.92, 1.54)	(0.88, 1.60)	(0.68, 2.22)	(0.56, 0.92)	(0.42, 0.80)	(0.52, 1.59)	(0.44, 0.99)	(0.14, 0.58)	(0.28, 2.14)
**9.5-12.7**	1.43*	1.57**	1.19	0.85	0.85	1.08	0.75	0.45*	2.66*
	(1.15, 1.78)	(1.22, 2.02)	(0.67, 2.10)	(0.67, 1.08)	(0.63, 1.13)	(0.62, 1.89)	(0.48, 1.18)	(0.23, 0.89)	(1.35, 5.22)
**> 12.7**	0.85	1.13	1.06	1.04	0.90	1.05	0.98	1.24	1.24
	(0.64, 1.14)	(0.82, 1.55)	(0.57, 1.97)	(0.83, 1.30)	(0.67, 1.21)	(0.58, 1.87)	(0.69, 1.38)	(0.84, 1.84)	(0.53, 2.91)

Adjusted for site, education and race.

***p < 0.0001.

**p < 0.001.

*p < 0.05.

Underweight women who gained less than 7.0 kg or between 9.5 and 12.7 kg during pregnancy had significantly increased odds of spontaneous preterm birth with PROM (OR = 1.70, 95% CI = 1.38-2.09) and without PROM (OR = 1.74, 95% CI = 1.36-2.22) compared to normal weight women. Similarly, the odds of spontaneous preterm birth with PROM (OR = 1.43, 95% CI = 1.15-1.78, and without PROM (OR = 1.57, 95% CI = 1.22-2.02) was higher among underweight women gaining 9.5-12.7 kg.

Among overweight and obese women, gaining less than 7.0 kg during pregnancy was statistically significantly associated with decreased odds of spontaneous preterm birth with and without PROM, compared to normal weight women. Further, gaining less weight (<7.0 kg) among overweight women was significantly associated with indicated (OR = 0.68, 95% CI = 0.41-0.99) compared to normal weight women.

Overweight and obese women who gained 7.0 to 9.4 kg during pregnancy had decreased odds of spontaneous preterm birth with or without PROM compared to normal weight women. Among overweight women, this weight gain was associated with nearly 30% decreased odds of spontaneous preterm birth with PROM (OR = 0.72, 95% CI-0.56-0.92) and over 40% decreased risk of spontaneous preterm birth without PROM (OR = 0.58, 95% CI-0.42-0.80). Obese women who gained 7.0 to 9.4 kg were also significantly less likely to have a spontaneous preterm birth with PROM (OR = 0.66, 95% CI = 0.44-0.99) and spontaneous preterm birth without PROM (OR = 0.28, 95% CI = 0.14-0.58) than normal weight women Further, obese women who gained 9.5-12.7 kg during pregnancy had lower odds of spontaneous preterm birth without PROM compared to normal weight women (OR = 0.45, 95% CI = 0.23-0.89). Additionally, obese women who gained 9.5-12.7 kg were 2.7 times more likely to have an indicated preterm birth compared to normal weight women (OR = 2.66, 95% CI = 1.35-5.22).

## Discussion

This study identified weight gain during pregnancy and pre-pregnancy overweight and obesity to have differential effects on spontaneous preterm birth with and without PROM and indicated preterm birth. While gaining less than 7.0 kg was found to be a risk factor for preterm birth with and without PROM among underweight women, it was a protective factor for women who were overweight and obese. Additionally, gaining 7.0-9.5 kg was associated with lower odds of spontaneous preterm birth with and without PROM. While gaining less than 7.0 kg was found to be protective among overweight women, gaining 9.5-12.7 kg was found to be a risk factor for indicated preterm births in obese women. Consistent to this analysis, several studies have found a positive relationship between obesity and preterm birth [4-6,14-17]. Zhong et al. in a recent study reported that being obese was associated with increased risk of preterm birth with PROM and decreased spontaneous preterm birth without PROM [23]. However, a number of studies failed to report a statistically significant association [11,13] and a study by Carnero et al. reported a negative association between obesity and spontaneous preterm birth [27]. This difference may be due to the inconsistency in controlling for confounding variables, variability in the definition of preterm birth and the lack of examination of weight gain during pregnancy as an effect modifier.

Although African American race is a known risk factor, racial differences in the association between gestational weight gain, obesity and preterm birth is not accentuated in this study. While gaining 7.0 kg was protective for preterm births with and without PROM, among both races, weight gain as high as 9.4 kg and 12.7 kg were found to be protective of preterm birth without PROM only in overweight and obese African American women, respectively. Few studies have also examined the racial differences between obesity and preterm birth [24,25]. However, none of these studies have examined racial differences between gestational weight gain, obesity and preterm birth.

Despite the inconsistencies in the literature, this study reported that, among obese women, gaining less weight is protective of spontaneous preterm birth with and without PROM. On the other hand, gaining excessive weight is a risk for indicated preterm birth. There is some evidence to suggest inflammatory reaction and infection related to obesity may be the biological mechanism that is responsible for the reported association between obesity and preterm birth [20,39,40]. Additionally, obesity is characterized by alterations of hypothalamic-pituitary-adrenal axis which is responsible for releasing metabolic hormones [41]. High level of corticotropin-releasing hormone (CRH) is a known risk factor for premature rupture of membranes, preterm labor, eclampsia and pregnancy-induced hypertension [42,43].

Prior studies had clearly established the relationship between obesity and fetal macrosomia [13,43]. Furthermore, historically, it was recommended to restrict weight gain during pregnancy, as a way to prevent complications associated with macrosomic infants [44]. However, later research reported that gaining little weight was associated with poorer infant survival rates [45]. It is only in recent years that researchers began focusing on the impact of excessive weight gain and the risk of preterm birth. This analysis has shed some light on the effect modification between pre-pregnancy weight and weight gain during pregnancy and its impact on preterm birth using data collected prior to the obesity epidemic. It is important to note that the findings from this study are on par with the current IOM guideline that recommended reduced weight gain during pregnancy for obese and overweight women [26]. The IOM guideline recommended that underweight (BMI < 18.5), normal weight (BMI = 18.5 – 24.9), overweight (BMI = 25.0-29.9), and obese (BMI ≥ 30) women should gain between 28-40 lbs, 25-35 lbs, 15-25 lbs, and 11-20 lbs, respectively.

The ability to utilize a large multisite data with over 45,000, ethnically diverse women is one of the major strengths of this study. Additionally, this dataset provided data on pre-pregnancy weight. Most available datasets do not provide data on pre-pregnancy weight and this dataset provided a unique opportunity to assess this association. This data allowed the evaluation of multiple confounding factors that may influence this association. However, other than race, maternal education, and study site, no other factors examined in this analysis showed a statistically significant confounding effect. Considering the data for this analysis was collected over 40 years ago, it is important to consider contextual differences such as advances in health care that could not be assessed in this analysis. Although obesity was not a major problem 40 years ago, this study has reported a significant association between obesity and preterm birth and the interaction between pre-pregnancy BMI and weight gain during pregnancy. Further analysis with more recent data, including an examination of the adherence to the current IOM guidelines, is warranted.

Despite its strengths, there were some limitations to this analysis. First, gestational weight gain was calculated by subtracting weight at first prenatal care from weight at delivery. This computation, did not take into account the duration of pregnancy. Calculating the weekly weight gain rate to adjust for the length of pregnancy was not possible due to insufficient data on the timing of the first prenatal care. Although calculating weight gain rate is a preferred method, it also ignores the normal pattern of weight gain during pregnancy, assuming a steadily constant weight gain throughout the pregnancy. Secondly, pre-pregnancy weight was self-reported and may be subject to recall bias. Third, gestational age was calculated by subtracting the last menstrual period from the date of delivery and may also be subject to recall bias. Fourth, the data had overrepresented inner city African American population which is not representative of the US population.

## Conclusions

In conclusion, this study reported that gaining less weight is protective of spontaneous preterm with and without PROM among obese and overweight women. Excessive weight gain during pregnancy among obese women is associated with indicated preterm birth. Health care professionals and public health workers should be aware of this risk and adhere to the 2009 IOM guideline.

## Prẻci

Pre-pregnancy overweight and Obesity coupled with excessive weight gain is a risk factor for early preterm birth.

## Competing interests

The authors declare that they have no competing interests.

## Authors’ contributions

SWM guided the analysis, wrote a significant portion of the manuscript. DLB participated in the data analysis, wrote a portion of the manuscript. MM participated in the analysis of the study. All authors read and approved the final manuscript.

## Pre-publication history

The pre-publication history for this paper can be accessed here:

http://www.biomedcentral.com/1471-2393/13/120/prepub
